# Persistent Organic Pollutant Exposure Leads to Insulin Resistance Syndrome

**DOI:** 10.1289/ehp.0901321

**Published:** 2009-11-19

**Authors:** Jérôme Ruzzin, Rasmus Petersen, Emmanuelle Meugnier, Lise Madsen, Erik-Jan Lock, Haldis Lillefosse, Tao Ma, Sandra Pesenti, Si Brask Sonne, Troels Torben Marstrand, Marian Kjellevold Malde, Zhen-Yu Du, Carine Chavey, Lluis Fajas, Anne-Katrine Lundebye, Christian Lehn Brand, Hubert Vidal, Karsten Kristiansen, Livar Frøyland

**Affiliations:** 1 National Institute of Nutrition and Seafood Research (NIFES), Bergen, Norway; 2 Department of Biochemistry and Molecular Biology, University of Southern Denmark, Odense, Denmark; 3 Department of Biology, University of Copenhagen, Copenhagen, Denmark; 4 INSERM U-870, INRA (Institut national de la recherche agronomique) U-1235, Lyon 1 University, Institut National des Sciences Appliquées de Lyon and and Hospices Civils de Lyon, Oullins, France; 5 The Bioinformatics Centre, Department of Biology and Biotech Research and Innovation Centre, University of Copenhagen, Copenhagen, Denmark; 6 Institut de Recherche en Cancérologie de Montpellier, INSERM U896 Metabolism and Cancer Laboratory, Montpellier, France; 7 Department of Insulin Pharmacology, Histology and Delivery, Novo Nordisk A/S, Maaloev, Denmark

**Keywords:** contaminants, farmed salmon, metabolic syndrome, nonalcoholic fatty liver, obesity, pollution, public health, type 2 diabetes

## Abstract

**Background:**

The incidence of the insulin resistance syndrome has increased at an alarming rate worldwide, creating a serious challenge to public health care in the 21st century. Recently, epidemiological studies have associated the prevalence of type 2 diabetes with elevated body burdens of persistent organic pollutants (POPs). However, experimental evidence demonstrating a causal link between POPs and the development of insulin resistance is lacking.

**Objective:**

We investigated whether exposure to POPs contributes to insulin resistance and metabolic disorders.

**Methods:**

Sprague-Dawley rats were exposed for 28 days to lipophilic POPs through the consumption of a high-fat diet containing either refined or crude fish oil obtained from farmed Atlantic salmon. In addition, differentiated adipocytes were exposed to several POP mixtures that mimicked the relative abundance of organic pollutants present in crude salmon oil. We measured body weight, whole-body insulin sensitivity, POP accumulation, lipid and glucose homeostasis, and gene expression and we performed microarray analysis.

**Results:**

Adult male rats exposed to crude, but not refined, salmon oil developed insulin resistance, abdominal obesity, and hepatosteatosis. The contribution of POPs to insulin resistance was confirmed in cultured adipocytes where POPs, especially organochlorine pesticides, led to robust inhibition of insulin action. Moreover, POPs induced down-regulation of insulin-induced gene-1 (*Insig-1*) and *Lpin1*, two master regulators of lipid homeostasis.

**Conclusion:**

Our findings provide evidence that exposure to POPs commonly present in food chains leads to insulin resistance and associated metabolic disorders.

Despite international agreements intended to limit the release of persistent organic pollutants (POPs) such as organochlorine pesticides, polychlorinated biphenyls (PCBs), polychlorinated dibenzo-*p*-dioxins (PCDDs), and polychlorinated dibenzofurans (PCDFs), POPs still persist in the environment and food chains (Atlas and Giam 1981; Dougherty et al. 2000; Fisher 1999; Jorgenson 2001; Schafer and Kegley 2002; Van den Berg 2009). Most human populations are exposed to POPs through consumption of fat-containing food such as fish, dairy products, and meat (Fisher 1999). Humans bioaccumulate these lipophilic and hydrophobic pollutants in fatty tissues for many years because POPs are highly resistant to metabolic degradation (Fisher 1999; Kiviranta et al. 2005). The physiological impact associated with chronic exposure to low doses of different mixtures of POPs is poorly understood, but epidemiological studies have reported that Americans, Europeans, and Asian patients with type 2 diabetes accumulated greater body burdens of POPs, including 2,3,7,8-tetrachlorodibenzo-*p*-dioxin (TCDD), 2,2′,4,4′,5,5′-hexachlorobiphenyl (PCB153), coplanar PCBs (PCB congeners 77, 81, 126, and 169), *p,p*′-diphenyldichloroethene (DDE), oxychlordane, and *trans*-nonachlor (Fierens et al. 2003; Henriksen et al. 1997; Lee et al. 2006; Rignell-Hydbom et al. 2007; Turyk et al. 2009; Wang et al. 2008).

The incidences of type 2 diabetes and the insulin resistance syndrome have increased at a globally alarming rate, and > 25% of adults in the United States have been estimated to be affected by metabolic abnormalities associated with insulin resistance (Ford et al. 2004). Impaired insulin action is a central dysfunction of the insulin resistance syndrome characterized by abdominal obesity and defects in both lipid and glucose homeostasis, increasing the risk for developing type 2 diabetes, cardiovascular diseases, nonalcoholic fatty liver disease, polycystic ovarian disease, and certain types of cancer (Biddinger and Kahn 2006; Reaven 2005). Although a sedentary lifestyle and consumption of high-fat food are considered major contributors to insulin resistance and obesity, these conventional risk factors can only partly explain the worldwide explosive prevalence of insulin resistance–associated metabolic diseases. We therefore sought to elucidate whether the exposure to POPs present in a food matrix could contribute to insulin resistance and metabolic disorders.

POPs accumulate in the lipid fraction of fish, and fish consumption represents a source of POP exposure to humans (Dougherty et al. 2000; Hites et al. 2004; Schafer and Kegley 2002). Therefore, certain European countries have dietary recommendations to limit the consumption of fatty fish per week (Scientific Advisory Committee on Nutrition 2004). On the other hand, n-3 polyunsaturated fatty acids present in fish oil have a wide range of beneficial effects (Jump 2002), including protection against high-fat (HF) diet–induced insulin resistance (Storlien et al. 1987). Accordingly, we fed rats an HF diet containing either crude (HFC) or refined (HFR) fish oil obtained from farmed Atlantic salmon and investigated the metabolic impacts of POPs and their ability to interfere with n-3 polyunsaturated fatty acids.

## Materials and Methods

Tissue RNA from liver of rats fed HFC and HFR was extracted using Trizol, and microarray analysis was performed using the Operon Rat Oparray. Levels of specific mRNA were quantified using real-time polymerase chain reaction (PCR) as described previously (Rome et al. 2008). 3T3-L1 cells were exposed to different POP mixtures, and we measured insulin-stimulated glucose uptake and mRNA expression of target genes. Details of the methods are available in the Supplemental Material (doi:10.1289/ehp.0901321).

### Animals

All experimental protocols described below were approved by the Norwegian State Board of Biological Experiments with Living Animals, and the animals were treated humanely and with regard for alleviation of suffering. Male Sprague-Dawley rats (Taconic, Ry, Denmark) weighing 200–250 g were housed with a 12-hr light/dark cycle and with free access to food and tap water. Animals were fed a standard diet (chow; 17% fat-derived calories, 3.4 kcal/g) or an HF diet (65% fat-derived calories, 5.5 kcal/g) for 28 days (Lavigne et al. 2001). Two additional HF diets were made by substituting corn oil (20% wt/wt) with either crude or refined salmon oil. Crude salmon oil was obtained by heating the rest raw material of farmed Atlantic salmon to 92°C and separating oil from water and solid material. Refined salmon oil was obtained by bleaching, carbon filtering, and deodorizing the crude oil. HF, HFC, and HFR diets were supplemented with cellulose (50 g/kg), choline bitartrate (2 g/kg), American Institute of Nutrition (AIN) vitamin mixture 76 (14 g/kg), and AIN mineral mixture 76 (67 g/kg) (MP Biochemicals, Inc, Illrich, France) to meet the daily nutrient requirement levels of adult rats (Reeves et al. 1993). Fatty acid composition of HF, HFC, and HFR diets was analyzed as previously described (Jordal et al. 2007).

### Hepatic lipids

We determined levels of triacylglycerol, diacylglycerol, and total cholesterol in frozen liver samples of overnight-fasted rats using high-performance thin-layer chromatography as described previously (Berntssen et al. 2005). Frozen (O.C.T. compound; Sakura Finetek Europe, Zoeterwoude, the Netherlands) and fixed (paraffin-embedded) liver sections were stained with Oil red O and hematoxylin and eosin (H&E), respectively.

### Determination of POP levels

We measured levels of POPs as described previously (Berntssen et al. 2005; Julshamn et al. 2004).

### Determination of insulin action in peripheral tissues

We used soleus muscles and epididymal fat of overnight-fasted animals to assess insulin-stimulated glucose uptake as described previously (Buren et al. 2002; Ruzzin et al. 2005).

### Hyperinsulinemic–euglycemic clamps

Animals were catheterized, and hyperinsulinemic–euglycemic clamps were performed 7 days later (Brand et al. 2003; Ruzzin et al. 2007). After a 6-hr fasting period, conscious unrestrained catheterized animals were infused with a prime (6 μCi) continuous (0.1 μCi/min for basal; 0.17 μCi/min for clamp) infusion of [3-^3^H]glucose from −90 to 120 min for assessment of whole-body glucose disappearance (*R*_d_) and appearance (*R*_a_) using Steele’s non–steady-state equations (Steele et al. 1956). The hyperinsulinemic–euglycemic clamp was performed (0–120 min) by a continuous infusion of human insulin (3 mU/kg/min) (Actrapid, Novo Nordisk, Bagsvaerd, Denmark), and euglycemia (~ 115 mg/dL) was maintained by variable infusion rates of a 20% nonlabeled glucose solution [glucose infusion rate (GIR)]. At the end of the clamp, rats were given a lethal dose of pentobarbital sodium; liver, epididymal fat, and gastrocnemius muscles were removed, frozen in liquid nitrogen, and stored at −80°C for determination of POP levels. Plasma glucose and insulin levels were analyzed by the glucose oxidase method (YSI 2300 STAT Plus glucose analyzer; YSI Incorporated, Yellow Spring, OH, USA) and an enzyme-linked immunosorbent assay kit (DRG Instruments, Marburg, Germany), respectively. To determine plasma [3-^3^H]glucose, plasma was deproteinized, dried to remove tritiated water, resuspended in water, and counted in biodegradable scintillation fluid (Nerliens Meszansky, Oslo, Norway) on a beta scintillation counter (Tri-Carb 1900TR; Packard, Meriden, CT, USA). All samples were run in duplicate. Hepatic glucose production (HGP) was calculated as tracer-determined *R*_a_ minus GIR.

Insulin resistance was further assessed by the homeostasis model assessment of insulin resistance (HOMA-IR) index as described by Lee et al. (2008).

### Cultured adipocyte studies

We used cultured and differentiated 3T3-L1 cells (Petersen et al. 2008) to assess insulin-stimulated glucose uptake and mRNA expression of target genes. On day 8 of the differentiation program, cells were exposed to vehicle (dimethyl sulfoxide) or POP mixtures for 48 hr, and glucose uptake was assessed.

### Cytotoxicity

Membrane integrity of POP-treated adipocytes was determined by the release of lactate dehydrogenase into cell medium by a Tox7 kit (Sigma-Aldrich, Leirdal, Norway).

### Statistical analysis

We examined differences between groups for statistical significance using analysis of variance (ANOVA) with the least-square difference post hoc test. We used one-class statistical analysis of microarray to identify differentially expressed genes (Tusher et al. 2001) between HFC- and HFR-fed rats. We determined statistical significance of the real-time PCR results using the Student’s *t*-test, and the threshold for significance was set at *p* ≤ 0.05.

## Results

### Characteristics of animals exposed to POPs

As we expected, concentrations of POPs were consistently much higher in the HFC diet than in the HFR diet [Supplemental Material, Table 1 (doi:10.1289/ehp.0901321)], whereas the contents of n-3 polyunsaturated fatty acids and other fatty acids were similar in the two diets because both the crude and the refined fish oils were obtained from the same batch of farmed salmon (Supplemental Material, Table 2 (doi:10.1289/ehp.0901321).

**Figure 1:**
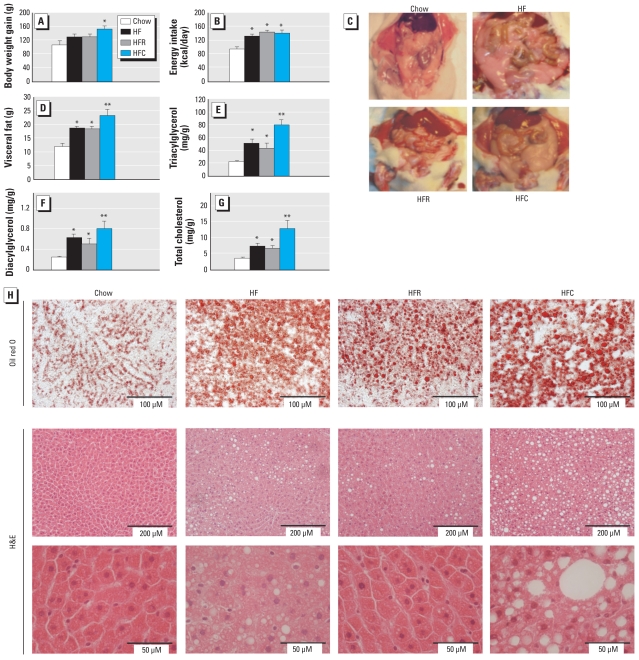
Characteristics of rats fed salmon oil containing POPs. Body weight gain (*A*) and daily energy intake (*B*) in rats fed chow or the HF, HFR, or HFC diets over a 4-week period. (*C*) Exposed ventral view of a representative rat from each diet group showing increased visceral adipose tissue after consumption of the HFC diet. (*D*) Quantification of visceral fat (epididymal and perirenal fat pads). (*E–G*) Levels of hepatic triacylglycerol (*E*), diacylglycerol (*F*), and total cholesterol (*G*). (*H*) Representative histological sections of liver stained with Oil red O (top) or H&E at low (middle) and high (bottom) magnifications; the three sections for each treatment group are from the same liver sample. All data are shown as mean ± SE; *n* = 8–9. *p < 0.02 compared with control. **p < 0.04 compared with HF.

After 28 days, rats fed the HFC diet appeared normal, although they tended to gain more weight than rats fed the HFR diet despite similar daily energy intake (Figure 1A, B). Intake of the HFC diet, but not HFR diet, enhanced the accumulation of visceral adipose tissue induced by HF consumption (Figure 1C, D). Profound dysregulation in lipid homeostasis was further observed in livers of HFC-fed rats, which exhibited elevated levels of triacylglycerol, diacylglycerol, and total cholesterol compared with HF-fed rats; livers of HFR-fed rats tended to exhibit a reduced lipid accumulation (Figure 1E–G). Histological examinations highlighted the development of severe hepatosteatosis in rats fed HFC (Figure 1H) and confirmed that the presence of POPs in salmon oil provokes significant impairment of lipid metabolism.

To gain further insight into the phenotypical changes of animals exposed to POPs, we performed a comparison of gene expression profiles in the liver of rats fed the HFC and HFR diets, using oligonucleotide microarrays. The expression of genes involved in drug metabolism was affected, indicating dietary POP exposure [Supplemental Material, Table 3 (doi:10.1289/ehp.0901321)]. We also observed major differences for genes involved in lipid metabolism and for several genes linked to lipid deposition (Supplemental Material, Table 3). Of interest, POPs induced robust down-regulation of insulin-induced gene-1 (*Insig-1*) and *Lpin1*, two master regulators of lipogenesis and synthesis of triglyceride and cholesterol (Croce et al. 2007; Engelking et al. 2004; Finck et al. 2006; Lee and Ye 2004). Real-time PCR analysis confirmed the strong repression of *Lpin 1* and *Insig-1* genes in the liver of rats consuming the HFC diet (Table 1). Similarly, in adipose tissue of HFC-fed rats, expression of *Lpin1* and *Insig-1* genes was repressed compared with HFR-fed animals [mean ± SE, 78 ± 8 vs. 55 ± 5 (*n* = 9, *p* = 0.02) for *Insig-1* and 98 ± 11 vs. 64 ± 8 (*n* = 9, *p* = 0.03) for *Lpin1* for HFR- and HFC-fed rats, respectively]. Furthermore, POPs induced a significant increase in the expression level of *SREBP1C* (sterol regulatory element-binding protein 1C), the master regulator of the lipogenic pathway, and *FAS* (fatty acid synthase), a well-known target gene of *SREBP1C* (Table 1). Interestingly, the hepatic expression of *LXR*α (liver X receptor alpha) was not affected, suggesting that the oxysterol pathway was not modified by POP exposure (Table 1). Altogether, these results demonstrate that POP exposure significantly affects the expression of critical genes involved in the regulation of lipid homeostasis. Gene set enrichment analysis further revealed significant effects on several biological pathways [Supplemental Material, Table 4 (doi:10.1289/ehp.0901321)]. This analysis demonstrated a highly significant up-regulation of pathways designated “pathogenic *Escherichia coli* infection” (EPEC/EHEC). The core genes up-regulated in the pathways include *TLR5*, *ROCK2*, *CD14*, and *YWHAZ*, a gene encoding a member of the 14-3-3 family of proteins reported to interact with insulin receptor substrate-1 and thereby regulating insulin signaling. Similarly, the roles of toll-like receptors, CD14, and rho kinases in regulating insulin signaling and establishment of insulin resistance in response to chronic low-grade inflammation are well documented (Begum et al. 2002; Cani et al. 2007; Furukawa et al. 2005; Petersen et al. 2008; Tzivion et al. 2001).

### Effects of POPs on insulin action *in vivo.*

**Figure 2:**
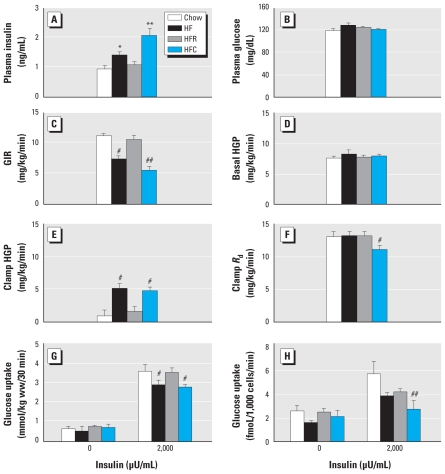
Effects of salmon oil and POPs on insulin action and glucose metabolism evaluated by hyperinsulinemic–euglycemic clamps performed in rats fed chow or HF, HFR, or HFC diets over a 4-week period. (*A*) Basal insulinemia. (*B*) Basal glycemia. (*C*) GIR. (*D*) Basal HGP. (*E*) HGP during the clamps. (*F*) Glucose disposal rate (*R*_d_). (*G*) Insulin-stimulated glucose uptake in soleus muscles. (*H*) Insulin-stimulated glucose uptake in primary adipocytes. All data are shown as mean ± SE; *n* = 6–9. *p < 0.04 compared with chow control. **p < 0.04 compared with HF. ^#^*p* < 0.05 compared with HFR. ^##^*p* < 0.03 compared with HF.

Next, we assessed the impacts of POPs on whole-body insulin action. In the basal state, intake of the HFC diet exacerbated the hyperinsulinemia induced by HF consumption, whereas animals fed HFR and control diets had similar plasma insulin levels (Figure 2A). Basal plasma glucose levels were similar in all groups (Figure 2B), but the HOMA-IR index was significantly increased in rats fed the HFC diet (7.1 for control rats, 11.2 for rats fed HF, 8.4 for rats fed HFR, and 15.5 for rats fed HFC; *p* < 0.04).

The performance of hyperinsulinemic–euglycemic clamp, the gold standard for investigating and quantifying insulin resistance (Kraegen et al. 1983), revealed that the consumption of the HFC diet aggravated HF-induced reduced GIR, whereas HFR-fed rats showed no impairment of insulin action compared with control rats (Figure 2C). Reduced GIR reflects decreased insulin-mediated suppression of HGP, reduced insulin-stimulated peripheral glucose disposal rates, or both. Analysis of these parameters revealed that basal HGP was similar in all groups (Figure 2D), whereas suppression of HGP by insulin was impaired in animals consuming both HFC and HF diets (Figure 2E). Moreover, intake of HFC led to impaired insulin-mediated glucose disposal in peripheral tissues, which mainly include skeletal muscles and adipose tissue (Figure 2F). To investigate this further, we determined the rates of glucose uptake in isolated soleus muscles and primary adipocytes. We found that insulin-stimulated glucose uptake was reduced to a similar extent in skeletal muscle of animals fed HFC and HF diets (Figure 2G). In contrast, rats fed the HFR diet were protected against muscle insulin resistance (Figure 2G). In adipose tissue, the ability of insulin to stimulate glucose uptake was impaired in both the HFR and HF groups, and this metabolic defect was worsened by the consumption of the HFC diet (Figure 2H). Thus, exposure to POPs present in HFC exacerbated the deleterious metabolic effects of HF and attenuated the protective effects of n-3 polyunsaturated fatty acids, which indicates that the presence of environmental organic contaminants may influence the outcomes of food and dietary products.

There is growing evidence that mitochondrial dysfunction contributes to insulin resistance (Lowell and Shulman 2005). To assess the impact of POPs on hepatic mitochondrial content, we measured mitochondrial DNA levels by quantitative polymerase chain reaction (qPCR), using primers specific for the *COXII* gene, and determined the ratio between mitochondrial DNA and nuclear DNA as previously validated (Bonnard et al. 2008). We found no apparent modification of the amount of mitochondrial DNA in the liver of the animals fed HFC (ratio *COXII*/*PPIA*, 1.1 ± 0.2 (mean ± SE) for rats fed HFR and 0.9 ± 0.1 for rats fed HFC, *p* = 0.189). However, despite this apparent lack of change in mitochondrial content, we observed significant reduction in the expression of several genes related to mitochondrial function, such as *PGC1*α (peroxisome proliferator- activated receptor gamma-coactivator-1 alpha), citrate synthase, medium-chain acyl CoA dehydrogenase, and *SDHA* (succinate dehydrogenase) (Table 1), indicating the presence of alterations in mitochondrial function and oxidative capacities in the liver of the rats exposed to POPs.

Analysis of POPs distribution in these animals revealed that whereas both liver and adipose tissue stored organochlorine pesticides, indicator PCBs, mono-*ortho-*substituted PCBs, and non–*ortho*-substituted PCBs, the liver preferentially retained PCDDs or PCDFs [Supplemental Material, Table 5 (doi:10.1289/ehp.0901321)].

### Effects of POPs on insulin action *in vitro.*

**Figure 3:**
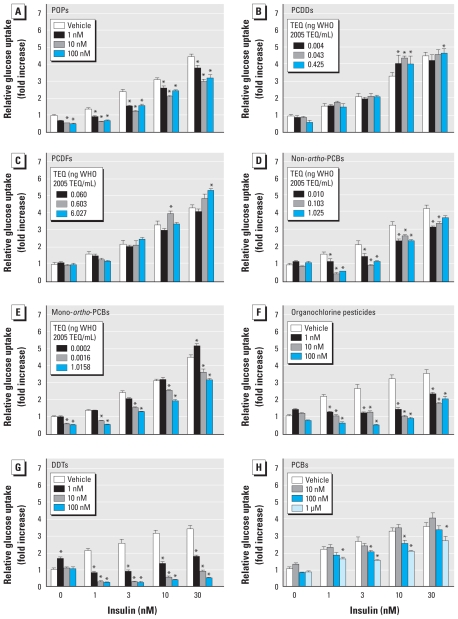
Effects of POPs on insulin action in adipocytes shown as the ability of differentiated 3T3-L1 adipocytes to take up radioactive-labeled glucose in response to insulin measured after 48 hr exposure to several POP mixtures found in crude oil from farmed Atlantic salmon. (*A*) POP mixture, (*B*) PCDD mixture, (*C*) PCDF mixture, (*D*) non–*ortho*-substituted PCB mixture, (*E*) mono–*ortho*-substituted PCB mixture, (*F*) Pesticide mixture, (*G*) DDT mixture, or (*H*) PCB mixture. Concentrations of POP mixtures are shown according to the highest contaminant compound present in the mixture, as well as the World Health Organization (WHO) 2005 TEQ for dioxins and dioxin-like PCBs (Van den Berg et al. 2006). Glucose uptake was determined in eight parallel wells for each mixture and for each concentration. Data are expressed as relative glucose uptake and presented as mean ± SE. *p < 0.05 compared with vehicle (dimethyl sulfoxide)-treated cells.

To further demonstrate the contribution of lipophilic POPs to the development of insulin resistance–associated metabolic disturbances, we exposed differentiated adipocytes to a POP mixture that mimicked the relative abundance of organic contaminants found in crude salmon oil. Incubation of adipocytes with this POP mixture impaired the ability of insulin to stimulate glucose uptake (Figure 3A), which is in agreement with the reduced insulin–stimulated glucose uptake observed in adipose tissue of rats fed the HFC diet (Figure 2H). We then determined whether POP exposure, as observed in rats fed the HFC diet, could affect the expression of *Lpin1* and *Insig-1* mRNA in cultured adipocytes. After 48-hr treatment with the POP mixture, *Lpin1* and *Insig-1* mRNA levels were dose-dependently reduced in adipocytes [Supplemental Material, Figure 1 (doi:10.1289/ehp.0901321)], which confirms the ability of POPs to interfere with key regulators of lipid metabolism. Importantly, the metabolic defects observed in adipocytes exposed to POPs were independent of cytotoxicity, as demonstrated by the absence of an increased release of lactate dehydrogenase into the cell culture media (Supplemental Material, Figure 2). Altogether, these findings clearly establish the capacity of POPs to impair insulin action and associated metabolic abnormalities in a cell-autonomous manner.

Humans and other organisms are chronically exposed to a variety of organic pollutants. To investigate which POPs contributed significantly to the impairment of insulin action, we incubated adipocytes with different POP mixtures. Although adipocytes exposed to a PCDD or PCDF mixture showed normal insulin action (Figure 3B, C), those exposed to non-*ortho*-substituted and mono-*ortho*-substituted PCB mixtures had reduced insulin action (Figure 3D, E). Impaired insulin action was independent of the total toxic equivalent (TEQ) concentration (Van den Berg et al. 2006) of the mixtures; up to 6.027ng WHO 2005 TEQ/mL for the PCDF mixture compared with 0.0016ng WHO 2005 TEQ/mL for the mono-*ortho*-PCB mixture. These findings demonstrate that risk assessment based on TEQ assigned to dioxins and dioxin-like PCBs (Van den Berg et al. 2006) is unlikely to reflect the risk of insulin resistance. Further investigations showed that insulin-stimulated glucose uptake was dramatically reduced in adipocytes treated with both the mixture of organochlorine pesticides (Figure 3F) and dichlorodiphenyltrichloroethanes (DDTs) (Figure 3G), whereas the mixture of indicator PCBs had less inhibitory effects on insulin action (Figure 3H).

## Discussion

In this study, we demonstrate for the first time a causal relationship between POPs and insulin resistance in rats. *In vivo*, chronic exposure to low doses of POPs commonly found in food chains induced severe impairment of whole-body insulin action and contributed to the development of abdominal obesity and hepatosteatosis. Treatment *in vitro* of differentiated adipocytes with nanomolar concentrations of POP mixtures mimicking those found in crude salmon oil induced a significant inhibition of insulin-dependent glucose uptake. These data provide compelling evidence that exposure to POPs increases the risk of developing insulin resistance and metabolic disorders.

Despite intense investigations and establishment of both preventive and therapeutic strategies, insulin resistance–associated metabolic diseases such as type 2 diabetes, obesity, and nonalcoholic fatty liver disease have reached alarming proportions worldwide (Angulo 2002; Ford et al. 2004; Zimmet et al. 2001). By 2015, the World Health Organization (WHO) estimates that > 1.5 billion people will be overweight and that 338 million people will die from chronic diseases such as diabetes and heart disease (WHO 2005). Although physical inactivity and regular intake of high-energy diets are recognized contributors (Hill and Peters 1998; Roberts and Barnard 2005), these lifestyle factors can only partially explain the explosive and uncontrolled global increase in metabolic diseases. Recently, the development of insulin resistance and inflammation was found to be exacerbated in humans and animals exposed to air pollution (Kelishadi et al. 2009; Sun et al. 2009). Furthermore, the widespread environmental contaminant bisphenol A was reported to impair pancreatic beta cells and trigger insulin resistance (Alonso-Magdalena et al. 2006). Our data, together with the finding that type 2 diabetics accumulate significant body burdens of POPs (Lee et al. 2006), provide additional evidence that global environmental pollution contributes to the epidemic of insulin resistance–associated metabolic diseases.

Although rats chronically fed the HFC diet for 28 days were exposed to a relatively high intake of organic pollutants, the concentrations of PCDDs/PCDFs and indicator PCBs in adipose tissue of these animals did not exceed those observed in Northern Europeans 40–50 years of age (Kiviranta et al. 2005), thereby indicating that doses of POP exposure sufficient to induce detrimental health effects were not excessive. Whether the exposure to lower levels of POPs would induce similar detrimental effects as those observed in the present study remains to be investigated.

Dietary interventions are current strategies to prevent or treat metabolic diseases, and nutritional guidelines are usually based on energy density and glycemic index of the diet; however, the levels of POPs present in food has received less attention. Given that POPs are ubiquitous in food chains (Fisher 1999), such underestimation may interfere with the expected beneficial effects of some dietary recommendations and lead to poor outcomes. For instance, the presence of POPs in food products may, to some extent, explain the conflicting results regarding the protective effects of n-3 polyunsaturated fatty acids against the incidence of myocardial infarction (Guallar et al. 1999; Rissanen et al. 2000). Overall, better understanding of the interactions between POPs and nutrients will help improve nutritional education of patients with insulin resistance syndrome.

To protect consumer health, the presence of contaminants in food is internationally regulated. In the European Union legislation, certain POPs including dioxins and dioxin-like PCBs are regulated in foodstuffs (European Union 2006). Risk assessment of these organic pollutants is based on the ability of individual compounds to produce heterogeneous toxic and biological effects through the binding of the aryl hydrocarbon receptor. Interestingly, we found that cultured adipocytes exposed to a PCDF or PCDD mixture have normal insulin action, even though the TEQ of these mixtures could be up to 3,500 times higher than the TEQ of the non-*ortho*-substituted and mono-*ortho*-substituted PCB mixtures that impaired insulin action. These findings demonstrate that risk assessment based on WHO TEQs assigned to dioxins and dioxin-like PCBs is unlikely to reflect the risk of insulin resistance and the possible development of metabolic disorders.

Although the production of organochlorine pesticides has been restricted since the 1970s, the global production and use of pesticides are poorly controlled (Jorgenson 2001; Nweke and Sanders 2009), and the presence of these environmental chemicals in seafood still remains unregulated in European countries (European Union 2008). Of the POP mixtures tested *in vitro*, organochlorine pesticides were the most potent disruptors of insulin action. This powerful inhibitory effect of pesticides on insulin action likely explains the common finding emerging from several independent cross-sectional studies reporting an association between type 2 diabetes and the body burdens of *p,p*′-DDE, oxychlordane, or *trans*-nonachlor (Lee et al. 2006; Rignell-Hydbom et al. 2007; Turyk et al. 2009). Therefore, widespread pesticide exposure to humans appears to be of particular global concern in relation to public health.

**Figure 4:**
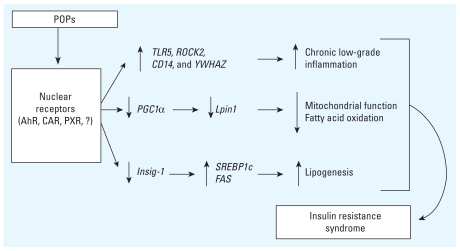
Schematic representation of the possible mechanisms behind the development of the insulin resistance syndrome induced by POP exposure. POPs may activate nuclear receptors including aryl hydrocarbon receptor (AhR), pregnane X receptor (PXR), constitutive androstane receptor (CAR), or yet unknown receptors. POP exposure may induce the regulation of genes involved in the inflammatory pathway, mitochondrial function, lipid oxidation, and lipogenesis, thereby contributing to the development of the insulin resistance syndrome.

We draw two main conclusions from these observations. First, exposure to POPs present in the environment and food chains are capable of causing insulin resistance and impair both lipid and glucose metabolism, thus supporting the notion that these chemicals are potential contributors to the rise in prevalence of insulin resistance and associated disorders (Figure 4). Second, although beneficial, the presence of n-3 polyunsaturated fatty acids in crude salmon oil (in the HFC diet) could not counteract the deleterious metabolic effects induced by POP exposure. Altogether, our data provide novel insights regarding the ability of POPs to mediate insulin resistance– associated metabolic abnormalities and provide solid evidence reinforcing the importance of international agreements to limit the release of POPs to minimize public health risks.

## Figures and Tables

**Table 1:** Real-time PCR determination of mRNA expression of a set of relevant genes in the liver of rats fed HFR or HFC diets (*n* = 9 per group).

	HFR	HFC	*p-*Value
Genes related to mitochondrial function			
*PGC1*α	0.73 ± 0.3	0.05 ± 0.02	0.043
*PPAR*α (peroxisome proliferator-activated receptor α)	76 ± 7	75 ± 18	0.988
*CS* (citrate synthase)	316 ± 19	214 ± 10	0.002
*SDHA* (succinate dehydrogenase)	74 ± 2	63 ± 4	0.038
*MCAD* (medium chain acyl CoA dehydrogenase)	332 ± 30	170 ± 18	0.003

Genes related to lipogenesis			
*SREBP1C*	3.0 ± 0.3	4.6 ± 0.6	0.021
*LXR*α	50 ± 3	51 ± 7	0.932
*FAS*	1.1 ± 0.1	1.9 ± 0.2	0.01
*Lpin 1*	96 ± 17	22 ± 10	0.0017
*Insig-1*	123 ± 23	43 ± 12	0.0071
